# Surgical mask-induced dyskinesia: a rare COVID-19 pandemics complication

**DOI:** 10.1055/s-0043-1771166

**Published:** 2023-07-26

**Authors:** Thiago Yoshinaga Tonholo Silva, Lucas de Oliveira Cantaruti Guida, José Luiz Pedroso, Orlando Graziani Povoas Barsottini

**Affiliations:** 1 Universidade Federal de São Paulo, Escola Paulista de Medicina, Departamento de Neurologia e Neurocirurgia, Setor de Neurologia Geral e Ataxias, Disciplina de Neurologia, São Paulo SP, Brazil.

A 64-year-old man presented with involuntary oromandibular movements since the start of the COVID-19 pandemics. Whenever the patient has worn a mask, he started the abnormal movements of the jaw, that promptly improved when he took off the mask ( Video 1 ). The patient was unaware of the movements and did not feel any urge to perform them, no tongue movement was observed, and there were no other relieving maneuvers. Apart from the oromandibular dyskinesia (OMD), neurological examination was normal.

**Video 1** Patient with involuntary and repetitive jaw movements while wearing a surgical mask. There is marked improvement of the movements when the mask is taken off. https://www.arquivosdeneuropsiquiatria.org/wp-content/uploads/2023/05/ANP-2022.0199-video.mp4


Surgical mask-induced dyskinesia phenomenology is uncertain, and somewhat similar to task-induced dystonia, a focal, isolated disorder that occurs only with specific actions. Although, it is thought to be choreic rather than dystonic, since the patients are not self-aware of the phenomenon. 1 Absence of use of antipsychotics and the exacerbation with sensory input help distinguishing from other forms of OMD. 1 2

**Figure 1:**
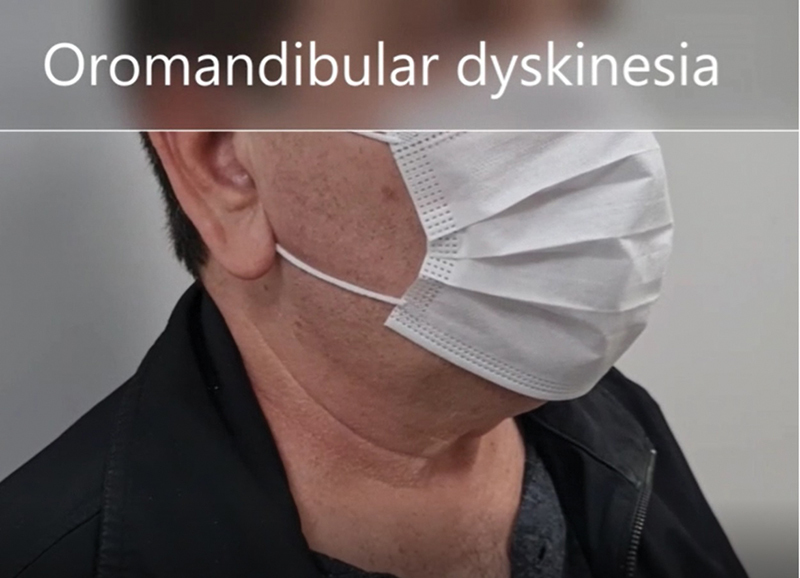
Oromandibular dyskinesia.
